# High Connectivity among Blue Crab (*Callinectes sapidus*) Populations in the Western South Atlantic

**DOI:** 10.1371/journal.pone.0153124

**Published:** 2016-04-11

**Authors:** Ana Luzia Figueiredo Lacerda, Ralf Kersanach, Maria Cristina Silva Cortinhas, Pedro Fernandes Sanmartin Prata, Luiz Felipe Cestari Dumont, Maíra Carneiro Proietti, Rodrigo Maggioni, Fernando D’Incao

**Affiliations:** 1 Instituto de Oceanografia, Universidade Federal do Rio Grande, Rio Grande do Sul, Brasil; 2 Instituto de Ciências Biológicas, Universidade Federal do Rio Grande, Rio Grande do Sul, Brasil; 3 Instituto de Ciências do Mar, Universidade Federal do Ceará, Ceará, Brasil; Duke University Marine Laboratory, UNITED STATES

## Abstract

Population connectivity in the blue crab *Callinectes sapidus* was evaluated along 740 km of the Western South Atlantic coast. Blue crabs are the most exploited portunid in Brazil. Despite their economic importance, few studies report their ecology or population structure. Here we sampled four estuarine areas in southern Brazil during winter 2013 and summer 2014 in order to evaluate diversity, gene flow and structure of these populations. Nine microsatellite markers were evaluated for 213 adult crabs, with identification of seven polymorphic loci and 183 alleles. Pairwise *F*_*ST*_ values indicated low population structure ranging from -0.00023 to 0.01755. A Mantel test revealed that the geographic distance does not influence genetic (r = -0.48), and structure/migration rates confirmed this, showing that even the populations located at the opposite extremities of our covered region presented low *F*_*ST*_ and exchanged migrants. These findings show that there is a significant amount of gene flow between blue crab populations in South Brazil, likely influenced by local current dynamics that allow the transport of a high number of larvae between estuaries. Considering the elevated gene flow, the populations can be considered a single genetic stock. However, further information on population size and dynamics, as well as fishery demands and impacts at different regions, are necessary for harvest management purposes.

## Introduction

Understanding the population structure of commercially valuable species is extremely important for identifying stocks, defining fishing boundaries, and managing exploitation of fishery resources [1,2]. The definition of limits for fisheries requires reliable information on gene flow and the number of migrants exchanged between different areas, since populations affected by natural or human pressures may or may not be reestablished by individuals from neighboring populations [2,3].

Genetic markers are commonly used to evaluate the degree of connection among populations of marine invertebrates, since the minute size of these organisms during their larval phase hinders direct observations of dispersal between areas [4,5]. Microsatellites are a type of genetic marker frequently applied in studies involving population and conservation genetics, and consist of tandem repetitions of short nucleotide motifs (2–6 bp) found abundantly in the genome [6,7]. Due to their high polymorphism, these markers are very useful in differentiating populations and inferring dispersal patterns [8–10]. For instance, microsatellites have been employed to better understand populations of animals with highly dispersive larvae such as the prawn *Penaeus monodon* [10] and the crab *Carcinus maenas* [11], as well the blue crab *Callinectes sapidus* [12]. This information can be applied to management strategies, since genetically structured populations should be considered separate management units for maintenance of genetic diversity [7].

The blue crab is a marine-estuarine crustacean [13,14] that lives for up to three and a half years [15]. Mating occurs inside estuaries and is closely coordinate with the molt cycle, which is controlled by temperature [16,17]. Gonadal maturation can occur at temperatures above 10°C, when females become active to forage and can therefore mature their ovaries [18]. Timing and duration of their spawning season is influenced by salinity, and therefore varies temporally and spatially [19]. In temperate areas with marked seasonality, spawning occurs during spring and peaks in summer, when salinity is usually higher [19,20]. At these zones, the reproductive cycle is characterized by copulation in estuarine waters, after which males remain in the upper estuary while inseminated females migrate to the high salinity waters of lower estuarine and shelf areas for egg deposition from the end of spring to the end of summer [16,19,20]. Larvae then hatch in the ocean, where they are influenced mainly by nearshore wind-generated surface currents. Eventually they return to the estuary through selective tidal stream transport identified using sensorial cues [21]. In this manner, during the larval phase blue crabs are subject to the oceanographic processes that occur in the coastal zone.

The eastern coast of Brazil in influenced by two main ocean current systems: the Brazil Current (BC) and the Malvinas Current (MC) [22]. The BC is formed at around 10°S, and is the western boundary current of the South Atlantic subtropical gyre, transporting warm, high salinity waters poleward and influencing most of the eastern coast during the entire year [23–25]. The MC is formed at around 55°S and flows northward carrying cold, low salinity waters, influencing mainly the South Brazilian coast in the winter when it is intensified [26,27]. The BC and MC interface at between 28–36°S in what is known as the Subtropical Confluence Zone; the latitude where this zone occurs can vary seasonally according to mass transport of both currents, as well as wind forcing [28]. These currents, as well as their seasonal variations, can influence regional transport of *C*. *sapidus* larvae.

Blue crabs represent an important commercial and recreational asset valued at approximately US$185 million worldwide in 2013 [29], and are the most exploited portunid species in Brazil [30]. In some regions, this species is commonly used as an alternative during closed seasons of other fishery resources, such as the anchovy *Anchoviella lepidentostole* in the southeast and the pink shrimp *Farfantepenaeus paulensis* in South Brazil, when it is commonly caught using banned fishing gear [30]. Despite its economic importance in the region, few studies discuss the ecology and populations of *C*. *sapidus* in the Western South Atlantic [31]. Such studies are extremely important for producing baseline data and establishing management strategies of blue crab fishery stocks.

In this context, the present work aimed to evaluate the genetic diversity and connectivity of blue crab populations in Western South Atlantic. Since coastal ocean currents are likely to influence larval transport and gene flow between areas, we tested the hypothesis that there are seasonal differences in gene flow between populations due to variations in currents.

## Materials and Methods

### Ethics statements

This work was approved by the evaluation committee of the Biological Oceanography Master Program of the Universidade Federal do Rio Grande-FURG. According to Normative Instruction 154/March 2007, all capture, tagging, sampling and transport of biological samples of wild animals for scientific purposes must have approval from Instituto Chico Mendes de Conservação da Biodiversidade (ICMBio) SISBIO committees. This study was approved by ICMBio, and conducted under SISBIO license #40765–1.

### Sampling

**Fig 1:**
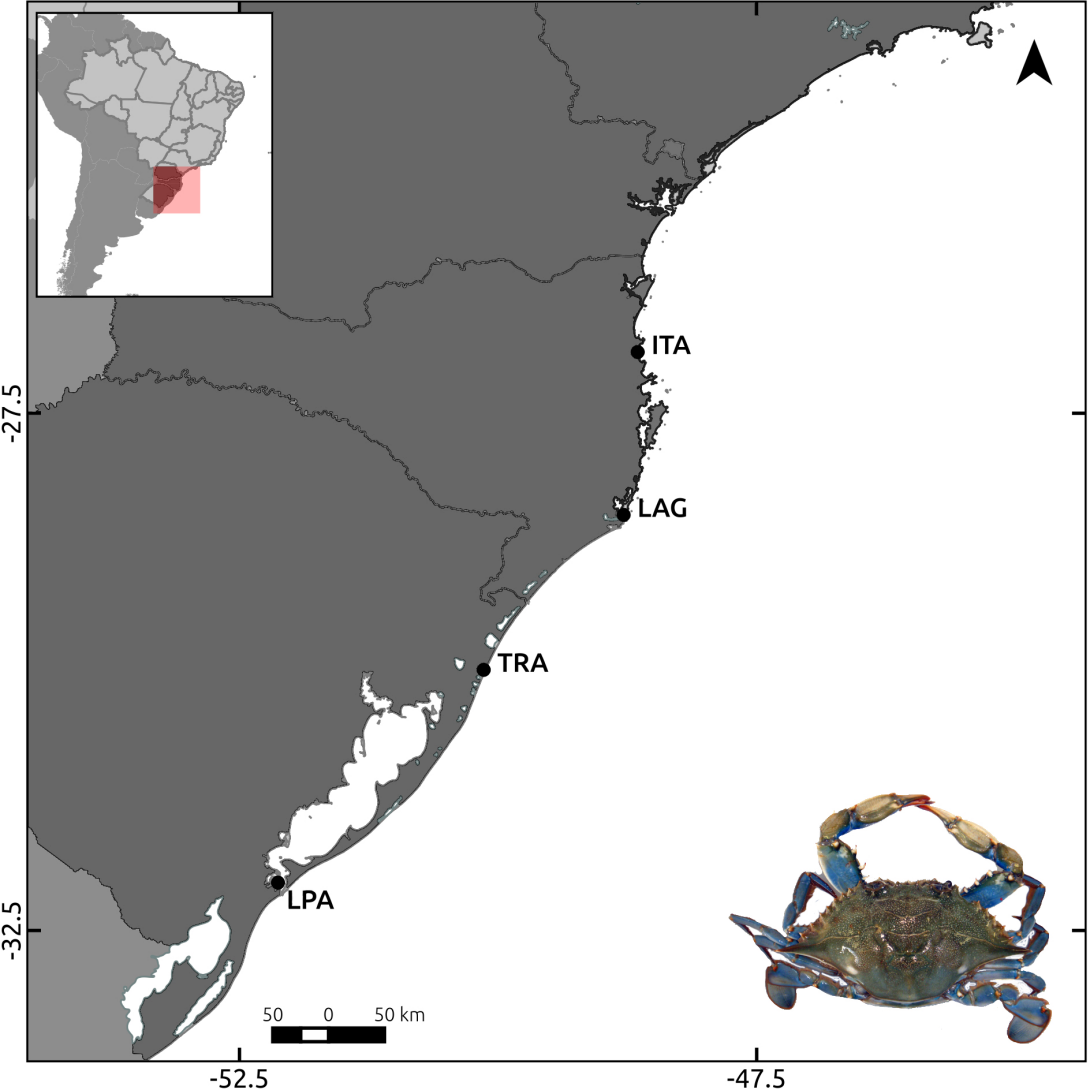
*Callinectes sapidus* sampling areas in the Western South Atlantic. LPA = Lagoa dos Patos; TRA = Tramandaí; LAG = Laguna; ITA = Itajaí.

Adult blue crabs were sampled along 740 km of the cost of Santa Catarina (SC) and Rio Grande do Sul (RS) states, in South Brazil, at four areas: Itajaí and Laguna (SC), Tramandaí and Lagoa dos Patos (RS) (Fig 1). Samples were collected in winter 2013 and summer 2014 at each location, but the Tramandaí samples from summer were not used since inadequate storing degraded their DNA. Geographical coordinates and sample size of each area are shown in Table 1.

**Table 1:** Coordinates and sample sizes of study areas in winter 2013 and summer 2014.

Location	Abbreviation	Latitude	Longitude	Sample size	
				Winter (W)	Summer (S)
**Lagoa dos Patos**	LPA	32°02’23.91”S	52°07’43.32”O	30	28
**Tramandaí**	TRA	29°58’56.29”S	50°08’22.69”O	34	-
**Laguna**	LAG	28°29’02.13”S	48°47’12.35”O	31	36
**Itajaí**	ITA	26°54’32.80”S	48°39’02.28”O	21	33

Individuals were captured using nets and traps at Lagoa dos Patos and Itajaí, or purchased directly from local fishermen after morphological identification at Tramandaí, Laguna and Itajaí. Sampling within regions was opportunistic, but always conducted inside the estuary in order to adequately represent the population. Muscle tissue was removed from the pereiopods, placed in absolute alcohol, and stored at -20°C temperature until laboratorial analyses.

### DNA extraction

Genomic DNA was extracted using standard proteinase K digestion and Phenol:Chlorophorm:Isoamyl Alcohol method [32]. Quantification and purity analysis were done with a NANODROP 2000® spectrophotometer, and DNA concentrations were standardized to 100 ng/μl using ultrapure water.

### Microsatellite amplification

Nine microsatellite loci previously described for *Callinectes sapidus* (12) were amplified through Polymerase Chain Reaction (PCR) (Table 2). Each PCR, contained: 10ng genomic DNA, 1.6 mM MgCl_2_, 30 mM Tris-HCL at pH 8.4, 75 mM KCL, 0.2 mM dNTP, 0.5 U Platinum Taq DNA polymerase (Invitrogen), 0.06 μM forward primer labeled with an M13 tail, 0.3 μM reverse primer, 0.24 μM universal fluorescent-label M13 primer, and ultrapure water to complete a total volume of 10 μL [33].

**Table 2:** Sequences and annealing temperature T (°C) of forward (F) and reverse (R) primers used in PCRs, with underlined sequence in M13 tail, as well as fluorescent dye labels used for genotyping.

Locus name	Primer sequence (5–3’)	Repeat motif	Anelling Temperature	Label
CSC-001	F: TGTAAAACGACGGCCAGTATTGGGTGGTTGCTTCAT	(CCTT)_14_	55°C	6-FAM™
	R: ACGAGGAGAAAGTTGAGATTGC			
CSC-004	F: TGTAAAACGACGGCCAGTACAACGGTAATTGTACGAGAAA	(TG)_16_	58°C	VIC^®^
	R: AGGCTAATGCCACCATCATC			
CSC-007	F: TGTAAAACGACGGCCAGTGGGACAAACAACATGAAAGTGG	(GA)_35_	59°C	PET^®^
	R: GAAAACCTATTCCGGGAAGC			
CSC-074	F: TGTAAAACGACGGCCAGTATGAGTACTGTGGCGTGTTTGG	(GT)_6_	60°C	VIC^®^
	R: CAAAGATGCCCCCTTATTTACC			
CSC-094	F: TGTAAAACGACGGCC AGTGTATCCACAACTGACTTTTCTCC	(TCTG)_6_	64°C	VIC^®^
	R: GGAGAAACACCCTCAGAAAACC			
CSA-035	F: TGTAAAACGACGGCC AGT GACTGGAGAAACGATAGGTG	(GT)_29_	46°C	NED™
	R: AACAAGGAGATTACACGGATTC			
CSA-073	F: TGTAAAACGACGGCC AGTGCCTATTTGCCTCGCTACCCC	(GT)_57_	55°C	NED™
	R:GTCACCAAAGTTGAGCAAGACTCTCT			
CSA-092	F: TGTAAAACGACGGCCAGTGTCAGTTTATTGGGAATCTCTTG	(GT)_13_	52°C	6-FAM™
	R: CTTCCATCCTAAACCACACCTGC			
CSA-121	F:TGTAAAACGACGGCCAGTAATAAGAGAACAAACACACGGGG	(AGAC)_9_	56°C	PET^®^
	R: AACTGCTTGCCTTCCTTCCATC			

Amplification conditions were the same for all primers, with variations only in the annealing temperature (Table 2). PCR conditions were: initial denaturation at 95°C for 2 min; 30 cycles of 1 min at 95°C, 30 s at 46–64°C (depending on the primer) and 1 min at 72°C; 8 cycles of 30 s at 94°C, 42 s at 53°C, and 1 min at 72°C; final extension step of 30 min at 72°C; and cooling to 4°C. Prior to genotyping PCR products were qualitatively analyzed through electrophoresis on 1% agarose gels stained with GelRed^TM^ (Biotium).

### Genotyping

Microsatellite loci were genotyped on an ABI 3500® (Applied Biosystems) capillary sequencer, and allele sizes obtained with the use of 600LIZ® internal size standard (Applied Biosystems). For genotyping optimization, samples labeled with different dyes were combined; therefore, each plate well contained PCR products of a same individual amplified with labeling dyes 6-FAM™, VIC®, NED™ and PET®. Results were visualized and molecular weight of alleles determined with GeneMapper v 4.1 (Applied Biosystems).

### Data analyses

Identification of null alleles and correction of allelic frequencies were performed with MICRO-CHECKER 2.2.3 [34]. Allele frequencies per locus, frequency of private alleles, observed and expected heterozygosis (respectively Ho and He), Hardy-Weinberg equilibrium (HWE) [35], linkage disequilibrium (LD), and inbreeding coefficient (*F*_*IS*_) [36], were obtained with GENEPOP 4.3 [37]. Significance of HWE was determined after a Bonferroni correction (α < 0.001).

Pairwise *F*_*ST*_ estimates and Analysis of Molecular Variance (AMOVA) were used to compare genetic differentiation among groups by season (winter and summer). All *F*_*ST*_ significances were determined after a Bonferroni correction (α < 0.001). Due to insignificant *F*_*ST*_ (α < 0.001) between seasons, winter and summer samples of each population were grouped and genetic structure inferred only between populations. Isolation by distance was evaluated by a Mantel test following Rousset [38] using the length of coastline between sampling sites and *F*_*ST*_ calculated across all loci. All pairwise *F*_*ST*_, AMOVA and the Mantel test were run in ARLEQUIN 3.0 [39].

Population clustering was done using a Bayesian approach in STRUCTURE 2.3.4 [40], assuming the ancestral model with population admixture, and correlated allele frequencies. Simulations were done using a 50,000 step burn-in, followed by 100,000 replicates of the Markov Chain Monte Carlo with the number of clusters varying from 1 to 4. For each K, twenty independent replicate runs were conducted in order to estimate ΔK [41], and then corrected in STRUCTURE HARVESTER to infer the most likely number of population clusters (K) though the Evanno method [42].

To estimate the migration rates between populations, a maximum likelihood approach based on the coalescent method was implemented in MIGRATE 3.6 [43] using default settings with Brownian motion approximation through mean values of the parameters θ and M, with *γ*_*ab*_ = θ_*b*_.M_*ab*_, where *γ* is the effective number of migrants, *θ* the populations size and M = *m*/*μ*, where *m* is the immigration rate and *μ* is mutation rate [43,44]. Migration rates are given as effective number of migrants.

## Results

### Allelic diversity

Seven of the nine analyzed microsatellite loci were polymorphic, displaying high levels of allelic diversity. Loci CSC-074 and CSA-092 were monomorphic in all locations, and were therefore excluded from all analyses. A total of 183 alleles were detected over the seven polymorphic loci at the four sampled areas, with a mean number of alleles per locus of 26.1. Locus CSA-073 presented the highest number of alleles (43 alleles) while locus CSA-121 displayed the lowest allelic diversity, with four alleles (Table 3).

**Table 3:** Number of alleles to same seven loci found in this study and in Steven *et al*., (2005), as well as private alleles in this study, separated by pairwise populations.

Locus		Number of alleles		Private alleles in this study (%)
	This study	Steven *et al*. 2005)	Location	(%)
CSA-121	4	5	LPA—TRA	0.0177
CSA-094	6	13	LPA—LAG	0.0153
CSA-001	24	42	LPA—ITA	0.0185
CSC-004	31	49	TRA—ITA	0.0210
CSC-035	33	64	TRA—LAG	0.0157
CSC-007	42	42	ITA—LAG	0.0132
CSC-073	43	48		
TOTAL	183	263	Average	0.0167		

LPA = Lagoa dos Patos; TRA = Tramandaí; LAG = Laguna; ITA = Itajaí.

Genetic variability was similar among samples. Two tetranucleotide microsatellite loci, CSA-121 and CSC-094, displayed lower allelic diversity than the remaining polymorphic loci. The five dinucleotide microsatellite loci (CSA-035, CSA-073, CSC-001, CSC-004, CSC-007) were highly polymorphic and all displayed private alleles to some populations. The frequency of private alleles between locations was low (Table 3).

### Heterozygosity and Hardy-Weinberg Equilibrium

Expected heterozygosity (He) per locus varied from 0.1374 (locus CSC-094 at Tramandaí) to 0.9558 (locus CSC-007 at Laguna), and observed heterozygosity (Ho) ranged from 0.0882 (locus CSC-094 at Tramandaí) to 0.9444 (locus CSA-073 at Itajaí). Mean expected heterozygosity of all loci was 0.5891, 0.4410, 0.5414 and 0.6148, and mean observed heterozygosity was 0.5419, 0.4538, 0.4392 and 0.5749 for Lagoa dos Patos, Tramandaí, Itajaí and Laguna, respectively. Loci CSC-007, CSC-004, CSC-001 and CSA-073 showed deviations from HWE. In this manner, among the 28 locus-area combinations (seven loci, four populations), significant deviation from HWE was observed for a total of eight groups (α < 0.001) (Table 4).

**Table 4:** Genetic diversity of seven nuclear loci of blue crabs populations sampled at the Western South Atlantic.

	Lagoa dos Patos	Tramandaí	Itajaí	Laguna
**CSA-121**				
*N*	57	33	47	67
*H*	4	3	4	4
*H*_*O*_	0.2069	0.2647	0.2037	0.1940
*H*_*E*_	0.2223	0.2412	0.3035	0.2524
*HWE*	0.0091	1.0000	0.0065	0.1133
*F*_*IS*_	-0.0247	-0.0646	0.2878	0.1040
**CSA-035**				
*N*	54	33	52	65
*H*	20	11	17	22
*H*_*O*_	0.6379	0.4706	0.6111	0.6269
*H*_*E*_	0.6806	0.5095	0.5877	0.6540
*HWE*	0.0196	0.2662	0.5659	0.1452
*F*_*IS*_	0.0244	0.0153	-0.0020	0.0097
**CSA-073**				
*N*	58	34	53	66
*H*	26	12	21	28
*H*_*O*_	0.8276	0.7941	0.9444	0.7705
*H*_*E*_	0.8024	0.7204	0.7663	0.7463
*HWE*	0.0840	0.0000*	0.0000*	0.0182
*F*_*IS*_	-0.0089	0.0186	-0.0363	0.0590
**CSC-001**				
*N*	56	24	40	67
*H*	13	7	9	16
*H*_*O*_	0.2413	0.1470	0.3333	0.3582
*H*_*E*_	0.5683	0.3679	0.5051	0.6041
*HWE*	0.0000*	0.0000*	0.0000*	0.0000*
*F*_*IS*_	0.5038	0.6771	0.03572	0.3936
**CSC-004**				
*N*	54	32	43	66
*H*	14	10	10	19
*H*_*O*_	0.8103	0.6470	0.6111	0.7611
*H*_*E*_	0.7270	0.7021	0.5869	0.7415
*HWE*	0.0021	0.0931	0.0000*	0.0757
*F*_*IS*_	0.0886	0.1737	0.1258	0.0963
**CSC-007**				
*N*	55	28	40	67
*H*	26	23	26	33
*H*_*O*_	0.8103	0.7647	0.7037	0.9253
*H*_*E*_	0.8843	0.7764	0.7034	0.9558
*HWE*	0.0000*	0.0516	0.0040	0.0073
*F*_*IS*_	0.0835	0.0301	0.0398	0.0476
**CSC-094**				
*N*	57	25	46	67
*H*	5	3	4	5
*H*_*O*_	0.2586	0.0882	0.2777	0.3880
*H*_*E*_	0.2383	0.1374	0.3370	0.3488
*HWE*	1.0000	0.2086	0.0706	0.9191
*F*_*IS*_	-0.0293	0.2420	0.2671	-0.0313

*N* number of genotypes, *h* number of haplotypes, *Ho* observed heterozygosity, *H*_*E*_ expected heterozygosity, *HWE* Hardy-Weinberg equilibrium and *F*_*IS*_ inbreeding coefficient. Asterisks indicate significant HWE values after a standard Bonferroni correction (*α < 0.001).

### Population structure and migration rates

The inbreeding coefficient (*F*_*IS*_) varied from -0.0646 at locus CSA-121 to 0.6771 at locus CSC-001 (both at Tramandaí), and mean *F*_*IS*_ for all areas was 0.1116 (Table 4). As noted above, when analyzing structure by season, no *F*_*ST*_ values were significant after the Bonferroni correction (α < 0.001) (Table 5). Therefore, although some variations were observed (slightly higher structure between populations in the winter than in the summer), individuals sampled in summer and winter were grouped per population for all remaining analyses.

**Table 5:** Pairwise *F*_*ST*_ values (below diagonal) and *p*-values (above diagonal) between locations, considering seasons (α < 0.001).

	LPA W	LPA S	TRA W	ITA W	ITA S	LAG W	LAG S
**LPA W**	-	0.4540	0.0173	0.0262	0.1634	0.1353	0.1336
**LPA S**	-0.00094	-	0.1745	0.2023	0.6583	0.4003	0.7399
**TRA W**	0.01939	0.00648	-	0.7355	0.0883	0.0131	0.0517
**ITA W**	0.01869	0.00501	-0.00788	-	0.0927	0.0433	0.1581
**ITA S**	0.00330	-0.00507	0.00702	0.00551	-	0.2830	0.4298
**LAG W**	0.00720	0.00020	0.02376	0.01832	0.00085	-	0.7924
**LAG S**	0.00598	-0.00436	0.01178	0.00532	-0.00119	-0.00489	-

Pairwise *F*_*ST*_ values varied from -0.00023 to 0.01755, and global *F*_*ST*_ was 0.00682, revealing very low structuring between the analyzed populations. Highest *F*_*ST*_ was observed between Tramandaí and Laguna (0.01755), which also indicates low, albeit significant (α < 0.001), differentiation (Table 6). The AMOVA brought additional evidence to support this low structure: variation was predominantly observed within populations (98%), with little variation among them (-0.08%). Correlation between geographic and genetic distances, as estimated with the Mantel test, was non-significant (r = -0.48).

**Table 6:** Pairwise *F*_*ST*_ values (below diagonal) and *p*-values (above diagonal) between locations.

	LPA	TRA	ITA	LAG
**LPA**	-	0.03605	0.14414	0.18919
**TRA**	0.01245	-	0.27027	0.00000*
**ITA**	0.00323	-0.00023	-	0.03604
**LAG**	0.00373	0.01755	0.00408	-

*Asterisks indicate significant values (*α < 0.001).

**Fig 2:**
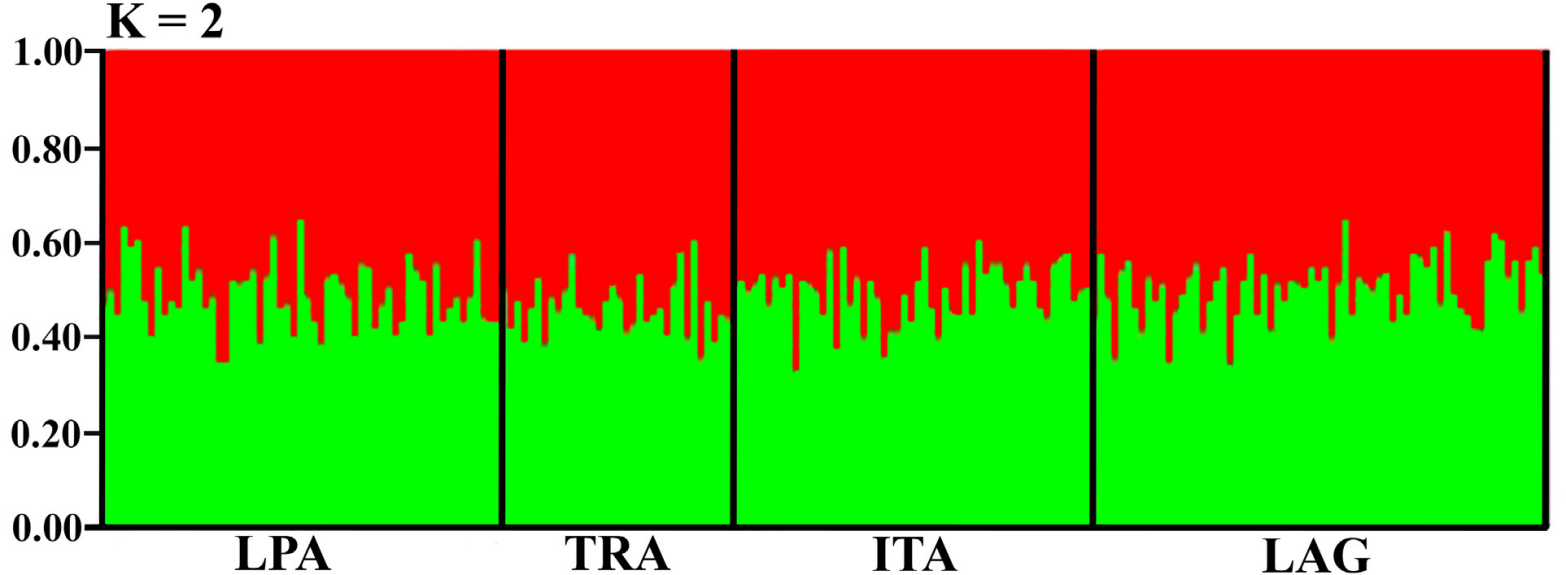
STRUCTURE assignment probabilities for blue crabs in South Brazil. K = 2 clusters. Each vertical bar represents one individual, and vertical black lines separate the sampled populations: LPA = Lagoa dos Patos; TRA = Tramandaí; ITA = Itajaí; LAG = Laguna.

Bayesian posterior probabilities indicated that the sampled populations were grouped into two clusters (K = 2), with all individuals presenting almost equal probabilities of being assigned to both clusters (Fig 2). This once again indicates a scenario of low structure and high admixture among blue crab populations in South Brazil.

**Fig 3:**
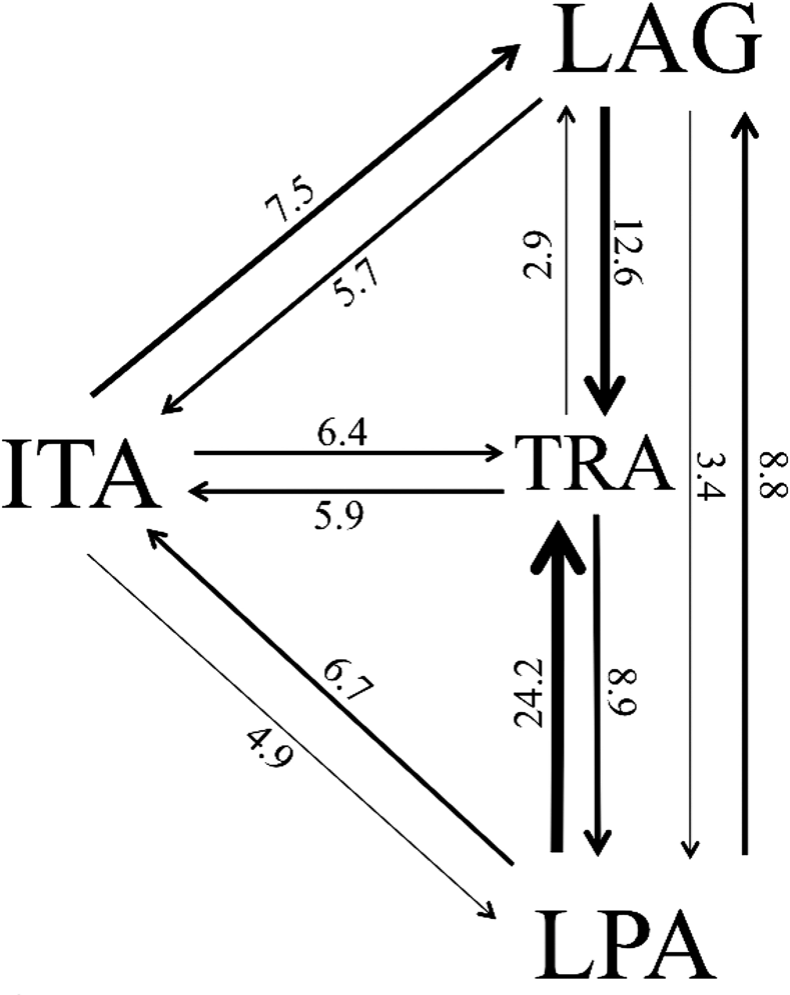
Directional migration rates between blue crab populations, given as effective number of migrants. LPA = Lagoa dos Patos; TRA = Tramandaí; ITA = Itajaí; LAG = Laguna.

Estimated migration rates showed highest number of migrants from Lagoa dos Patos to Tramandaí (*γ* = 24.19), and lowest from Tramandaí to Itajaí (*γ* = 2.90) (Fig 3). Tramandaí presented the hightes number of immigrants and Itajaí the lowest. The second lowest migration rate (*γ* = 3.44) was observed from Lagoa dos Patos to Itajaí, wich are the two populations located at the extremities of our study area range, separated by 740 km.

## Discussion

### Genetic diversity and HWE

*Callinectes sapidus* from four populations in the Western South Atlantic presented relatively high genetic diversity, with 183 alleles found in 213 individuals over the seven considered loci. However, this diversity was lower than the one reported by Steven *et al*. [12] for blue crabs from two locations of Chesapeake Bay, in the Western North Atlantic: 263 alleles in 134 individuals over the same seven loci. Heterozygosity and allelic diversity was also lower than those found by Steven *et al*. [12], as well as those reported for *Callinectes danae* at the same region studied in the present work [45]. Deviations from HWE observed in several groups indicate significant inbreeding. Elevated inbreeding was also found by Weber & Levy [45] for *Callinectes danae* in southern Brazil through allozymes analysis. Lower diversity and heterozygosity could be a result of continuous gene flow due to larval dispersal between areas, leading to shallow structure and admixture of *C*. *sapidus* populations at the studied region.

### Population structure and gene flow

Overall differentiation between blue crab populations in southern Brazil was very low with *F*_*ST*_ = 0.00682. This estimate is below the *F*_*ST*_ value reported for other *C*. *sapidus* populations in the Atlantic, even those separated by smaller distances than the 740 km considered in this work. Yednock *et al*. [2] analyzed blue crabs sampled along 300 km of the Gulf of Mexico, and found an overall *F*_*ST*_ of 0.09460, indicating a higher structure at this region when compared to the Western South Atlantic. Low structure and high gene flow was also shown through Bayesian clustering, number of migrants between populations, and AMOVA results, allowing us to infer a certain degree of panmixia among populations [3]. Pairwise comparisons between our sampled locations showed highest *F*_*ST*_ between Tramandaí and Laguna (0.01755), wich despite being significant (α < 0.001) is still quite low. Lagoa dos Patos and Itajaí, located at the two margins of our studied region, were not significantly structured (*F*_*ST*_ = 0.00373). These areas are separated by over 700 km and it would be expected that gene flow between them be limited, since migration is generally higher between geographically proximal areas [46]. However, gene exchange between marine populations is influenced by several factors other than distance, such as physical/hydrographical barriers, population sizes, life history patters, and prevailing ocean currents [3,47,48].

### Influence of currents on dispersal

Blue crab life history includes an oceanic larval period with seven or eight zoea followed by a megalopa phase, lasting around 31–69 days [13,49]. Therefore, this stage has high potential for dispersal through surface currents. Two large-scale currents possibly influence gene flow observed between the studies populations: the Brazil current, which courses poleward and is intensified in the summer; and the Malvinas Current, equator-bound and more intense in winter [27]. Coastal currents are a result of the impact of ocean currents on the topographical features of the continental shelf, also under strong influence of wind systems [50]. They act along the shelf in a seasonal manner and likely also influence larval dispersal between populations at the region. For instance, based on direction and speed of currents at the southern coast of Brazil, D’Incao [51] concluded that *Penaeus paulensis* (currently *Farfantepenaeus paulensis*) shrimp post-larvae disperse from the coast of Santa Catarina to Rio Grande do Sul from the end of winter to the end of summer. The same process could be responsible for *C*. *sapidus* larval transport between these areas, since blue crab females lay eggs in the open ocean from the end of spring to autumn [52]. This could explain the large number of migrants from Laguna to Tramandaí (see Fig 3).

The dynamic behavior of the Lagoa dos Patos plume can also influence larval flow along South Brazil. Marques *et al*. [53] showed that predominant southwestern (SW) winds during the winter increase the plume size along the coast and lead to northwards dispersal of organisms. During this season, this intensified plume can influence other lagoons and estuaries along the southern coast, likely transporting *C*. *sapidus* larvae from Rio Grande do Sul to Santa Catarina. Rodrigues *et al*. [52] found ovigerous females at the Lagoa dos Patos mouth at the end of spring, during summer, and in autumn. Considering the larval phase duration of this species, it is possible to infer that during winter the Lagoa dos Patos plume transports *C*. *sapidus* larvae ecloded at the end of summer and autumn to estuaries located northwards, reinforced by southwestern winds and the Malvinas Current [53]. This could explain the large number of migrants from this estuary to Tramandaí. In this manner, these surface ocean currents likely favor the extensive gene flow and consequent low structure of *Callinectes sapidus* populations along the southern coast of Brasil.

### Implications for management

This work is the first to infer, through genetic markers, the population structure of blue crab populations along the Southern Brazil coast. The results obtained in this work clearly show that *Callinectes sapidus* populations are highly connected throughout this region. High gene flow and low population structure were evidenced and attributed to dispersal by coastal currents, which present different directions and intensities according to season and likely transport blue crab larvae between areas. Information on stock structure is fundamental for determining impacts of exploitation on genetically separate stocks, and consequently establishing effective fisheries management policies. Considering the elevated gene flow between the blue crab populations along southern Brazil, they can be considered a single genetic stock. However, further information on population size and dynamics, as well as fishery demands and impacts at different regions, are necessary for harvest management purposes.
